# Supplementary material for: A new opportunity for the emerging tellurium semiconductor: making resistive switching devices
Source: Nat Commun. 2021 Oct 19;12:6081. doi: 10.1038/s41467-021-26399-1 (PMC8526830; doi:10.1038/s41467-021-26399-1)
Supplement: Supplementary file 1 — Supplementary Information [file 41467_2021_26399_MOESM1_ESM.pdf]

SUPPLEMENTARY INFORMATION

A new opportunity for the emerging tellurium semiconductor: making resistive switching devices

Y. Yang et al.

*Email: li_huanglong@mail.tsinghua.edu.cn

Supplementary figures and notes

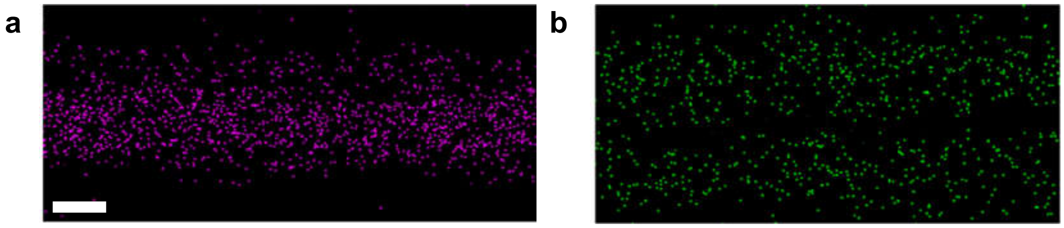

Supplementary figure 1 EDS elemental mapping images (scale bar: 20 nm) of the device cross-section area where (a) Sb is denoted in purple (a) and Te in green.

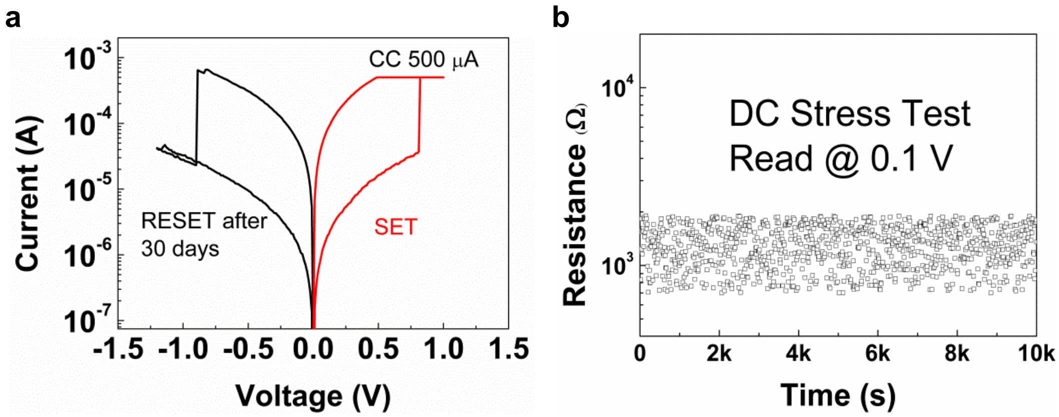

Supplementary figure 2 (a) RESET behavior of a Te/Sb_2Te_3/Te (TST) device 30 days after SET operation. (b) DC stress test of a TST device after SET operation for 10^4 seconds under constantly applied 0.1 V read voltage.

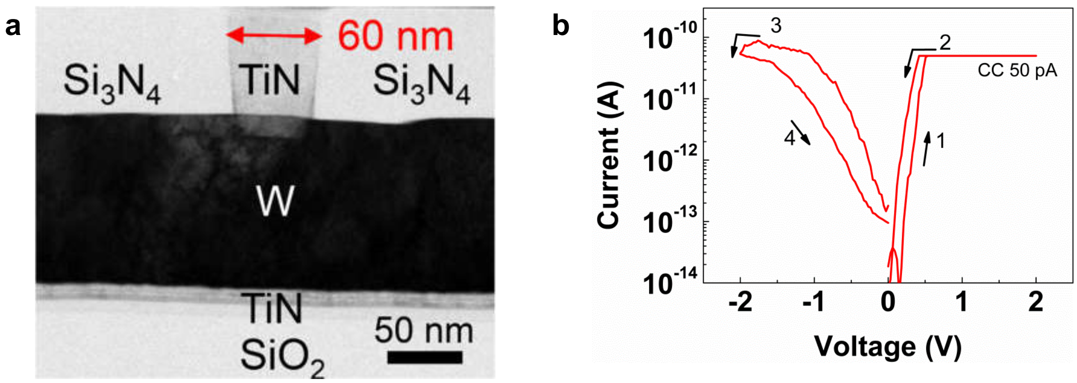

Supplementary figure 3 (a) TEM image of the 60-nm TiN bottom protective electrode. (b) NV-RS behavior of the 60-nm TiN/Te/Sb_2Te_3/Te/TiN (T'TSTT') device under 50-pA compliance current.

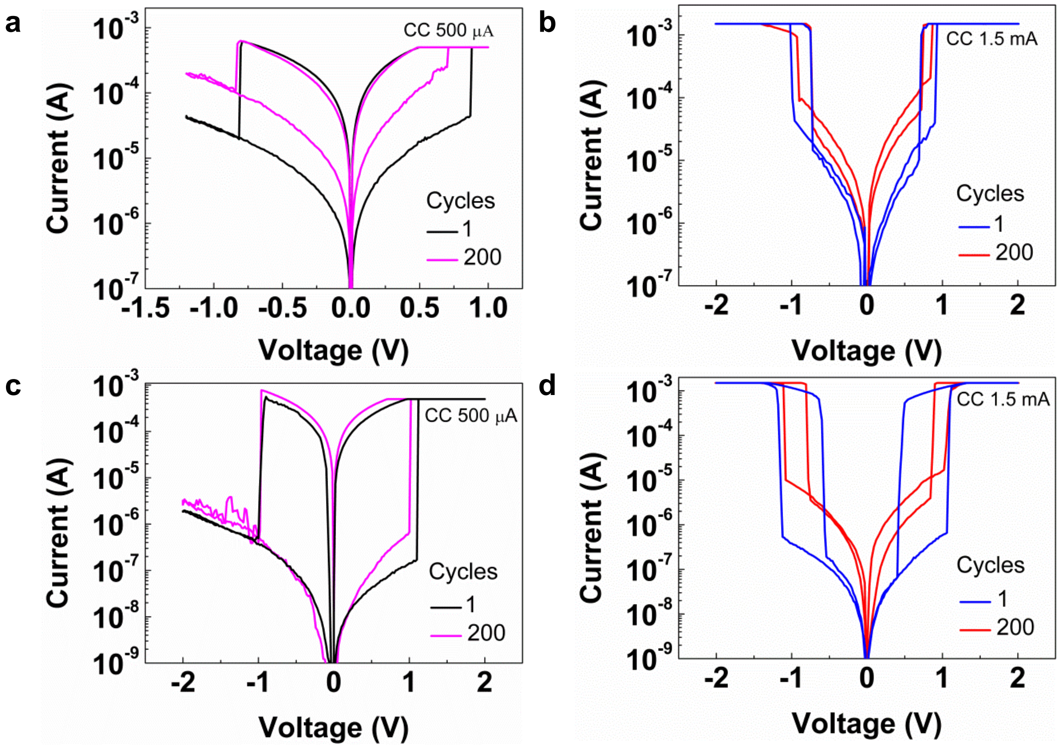

Supplementary figure 4 (a) DC I-V curves of the Pt protected TST (PTSTP) device obtained in the first and 200th cyclic voltage sweep under 500-μA CC. (b) DC I-V curves of the PTSTP device obtained in the first and 200th cyclic voltage sweep under 1.5-mA CC. (c) DC I-V curves of the Gd protected TST (GTSTG) device obtained in the first and 200th cyclic voltage sweep under 500-μA CC. (d) DC I-V curves of the GTSTG device obtained in the first and 200th cyclic voltage sweep under 1.5-mA CC.

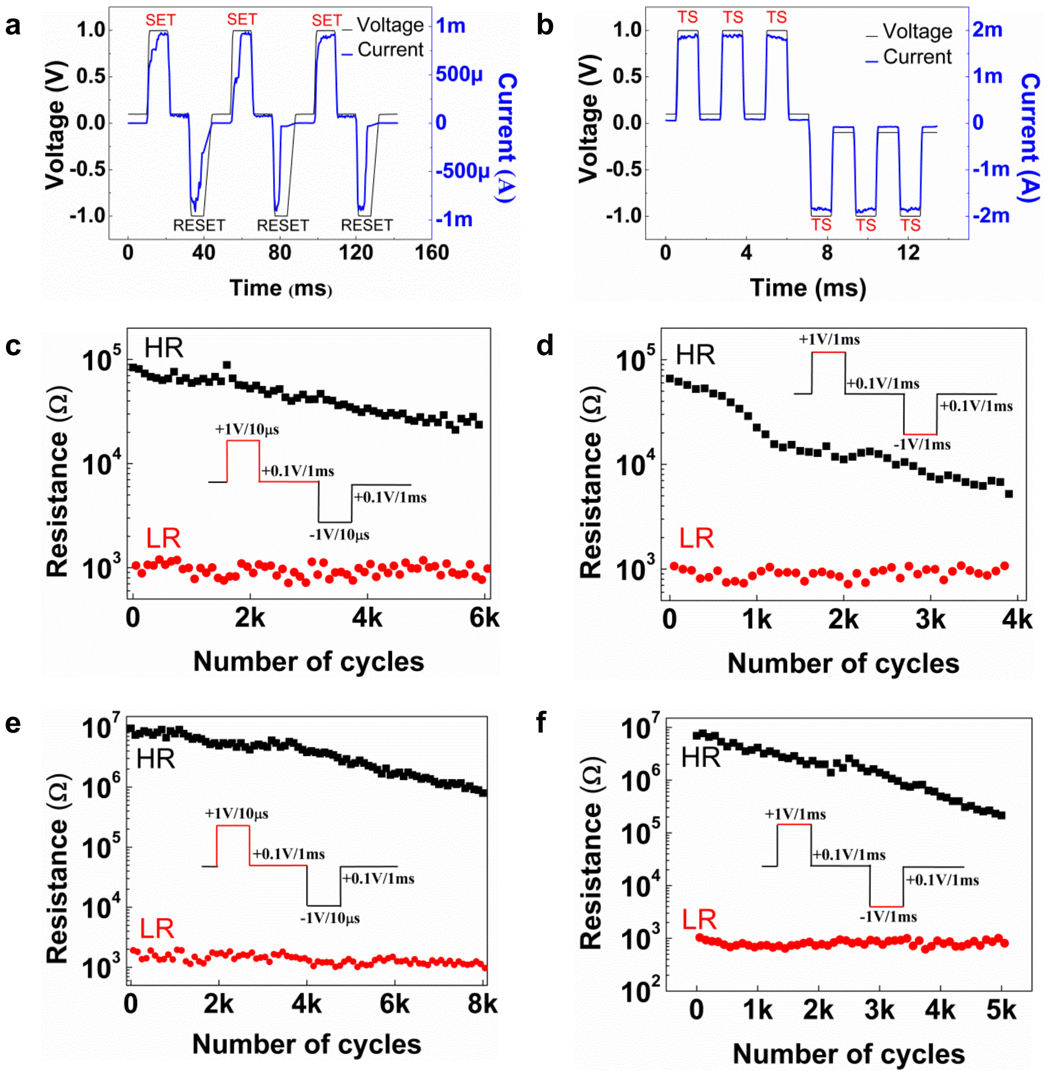

Supplementary figure 5 (a) Pulse train measurement with write pulse width of 10 µs and amplitude of ±1 V to achieve NV-RS operation in a PTSTP device. (b) Pulse train measurement with write pulse width of 1 ms and amplitude of ±1 V to achieve V-RS operation in a PTSTP device. (c) Endurance test of the PTSTP device in its NV-RS mode using a train of pulses (1-V amplitude and 10-µs width) with alternating polarities. (d) Endurance test of the PTSTP device in its V-RS mode using a train of pulses (1-V amplitude and 1-ms width) with alternating polarities. (e) Endurance test of the GTSTG device in its NV-RS mode using a train of pulses (1-V amplitude and 10-µs width) with alternating polarities. (f) Endurance test of the GTSTG device in its V-RS mode using a train of pulses (1-V amplitude and 1-ms width) with alternating polarities.

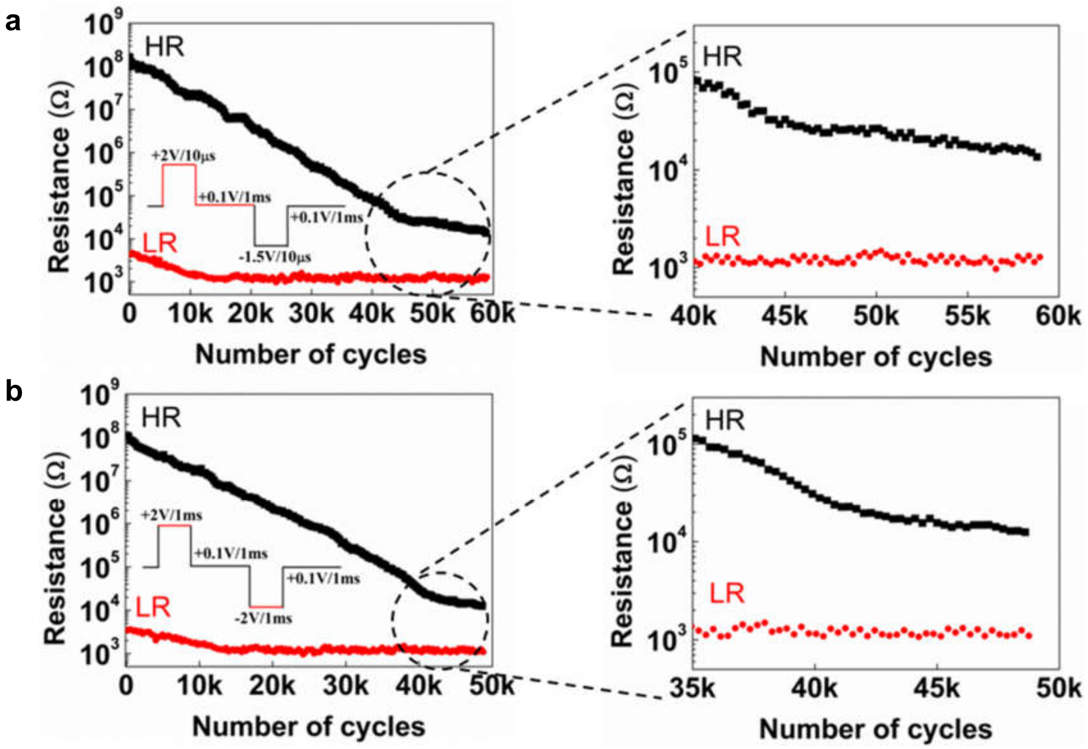

Supplementary figure 6 (a) Endurance test of the 150-nm T'TSTT' device in its NV-RS mode using a train of pulses (1.5-2.0-V amplitude and 10-µs width) with alternating polarities. (b) Endurance test of the T'TSTT' device in its V-RS mode using a train of pulses (2-V amplitude and 1-ms width) with alternating polarities. The HRs of the device in its NV-RS and V-RS modes degrade to values that are comparable to the initial ones of the 2-µm PTSTP devices after 40k and 35k cycles, respectively, from which HR degradation can be directly compared between these two devices.

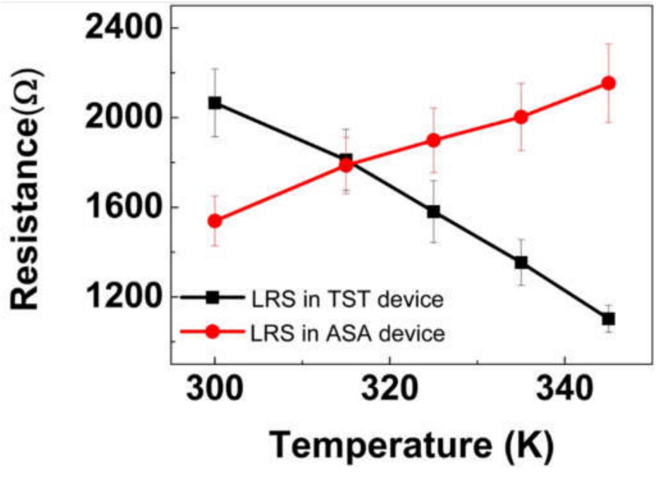

Supplementary figure 7 Temperature dependent ON-state resistances of Te/Sb_2Te_3/Te (TST) and Ag/Sb_2Te_3/Ag (ASA) devices. The temperature range is chosen so that Sb_2Te_3 does not undergo phase change.

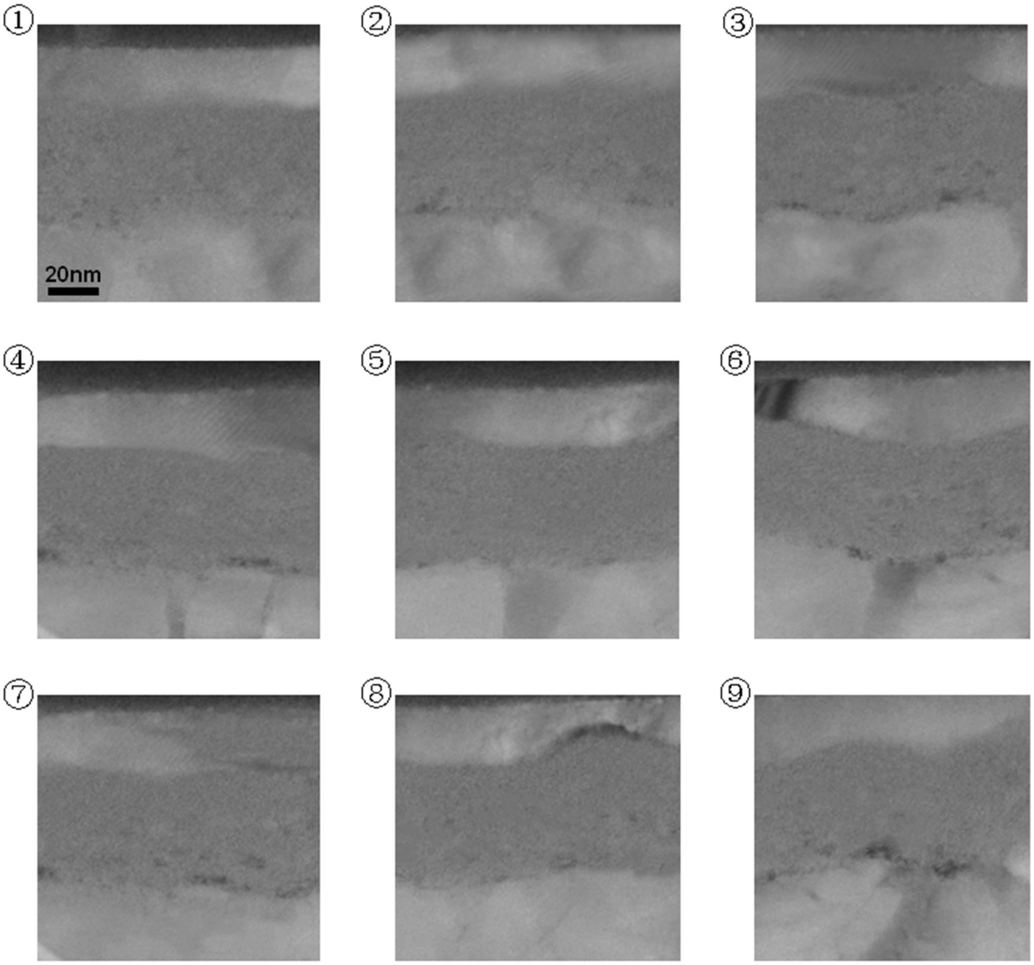

Supplementary figure 8 Nine unbiasedly-selected cross-sectional TEM images of as-fabricated TST devices from extensive characterizations where no filament-like structure has been found.

Supplementary note 1: Admittedly, the performed characterizations are not exhaustive, though extensive. Nevertheless, the several facts, including the absence of any filament-like feature in our extensively searched cross-sectional areas in the pristine devices, the electrode size-independent resistance of the SET switched device (figure 2a), the semiconducting property of the filament material (supplementary figure 7) and the lack of other conceivable (semi)conducting filament compositions than Te, still strongly support the Te filament switching mechanism of our device.

In the future works, in-situ characterizations of the electrochemical dynamics of nanoscale Te inclusions in various dielectrics with different Te^{2-} (or Te) diffusivity in lateral devices may provide more direct evidence of Te filament-based RS switching mechanism as well as scientific insights into the different electrochemical dynamics of the semiconducting Te inclusions compared to those of the metallic ones at the nanoscale[1].

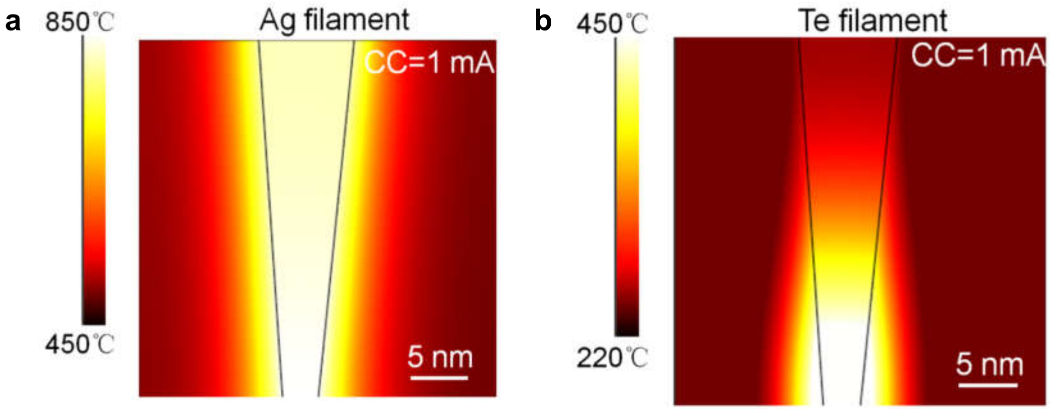

Supplementary figure 9 COMSOL thermodynamics simulations of the temperature distributions in (a) Ag filament-based device and (b) Te filament-based device. Filaments are modelled as inverted truncated cone-shaped structures of the same physical dimensions (30 nm in height, 8 nm and 3 nm in diameter for the top and bottom surfaces, respectively)

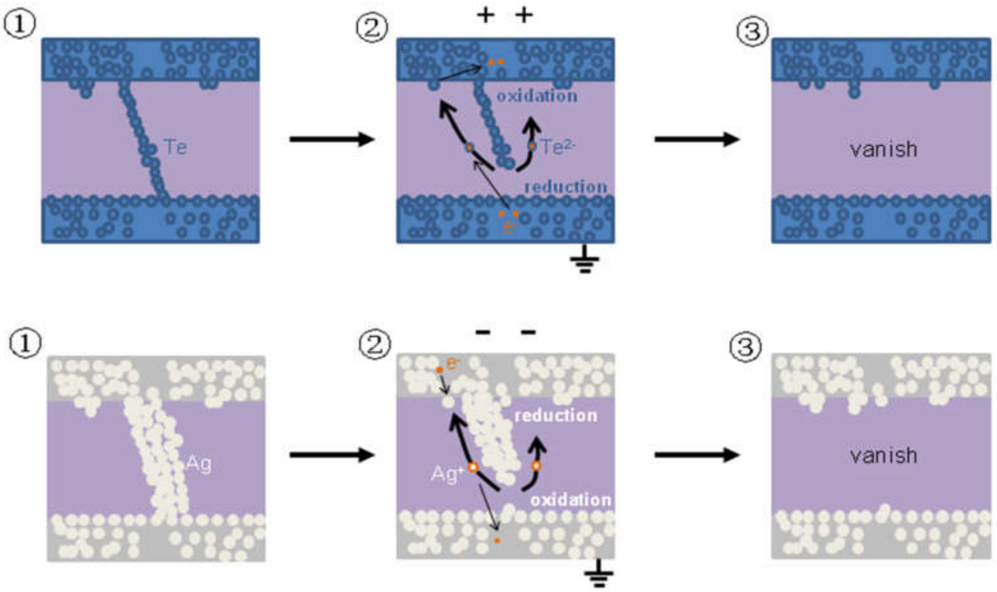

Supplementary figure 10 Schematic comparison of the RESET processes between Te filament-based device and Ag filament-based device in their NV-RS modes.

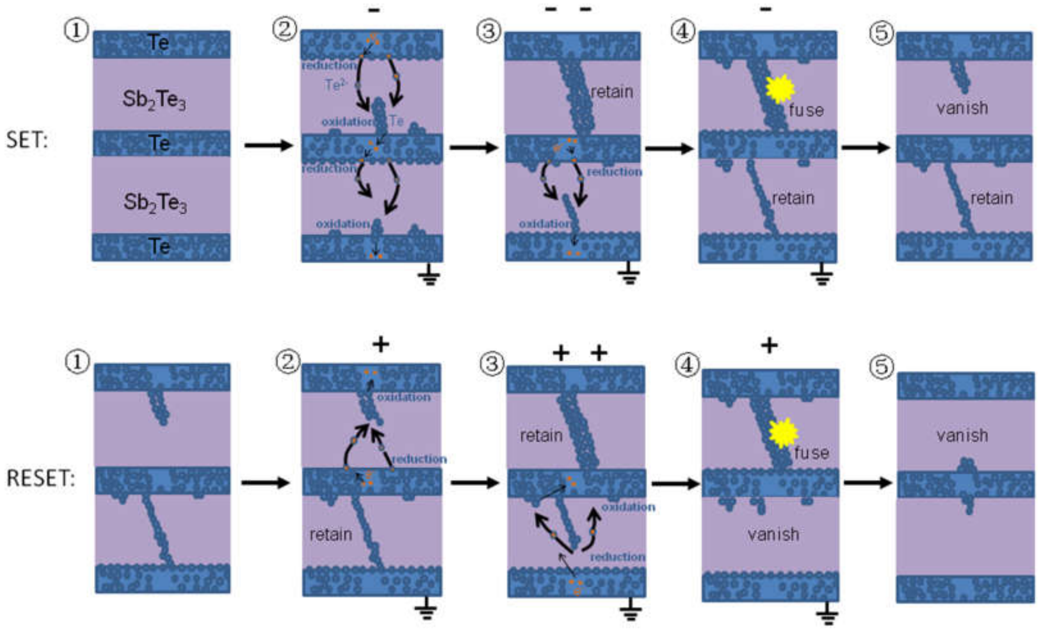

Supplementary figure 11 Schematic of the RS process in the TSTST device.

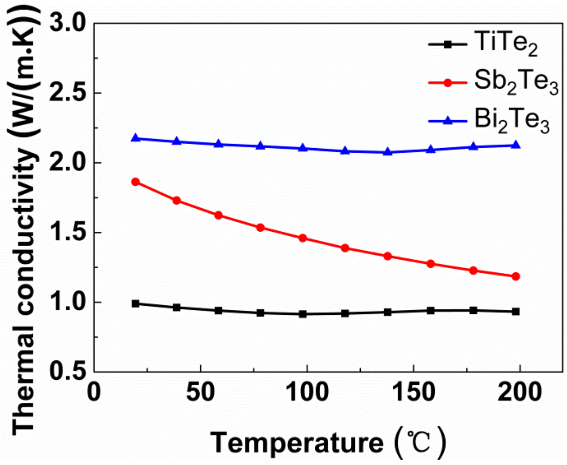

Supplementary figure 12 Thermal conductivities of 200-nm-thick $TiTe_2$, Sb_2Te_3 and Bi_2Te_3 films measured by 3-ω method. All the samples are deposited on 100-nm-thick Te layers and the thermal conductivity of thin-film Te as the baseline value has been deducted.

Reference

[1] Yang, Y., Gao, P., Li, L., Pan, X. Q., Tappertzhofen, S., Choi, S. H., Waser, R., Valov, I. & Lu, W. D. Electrochemical dynamics of nanoscale metallic inclusions in dielectrics. *Nat. Commun.* **5**, 4232 (2014).
